# Automated capture management failure leading to ventricular fibrillation: Is automatic capture management for all or personalized pacing programming?

**DOI:** 10.1016/j.hrcr.2026.03.026

**Published:** 2026-04-07

**Authors:** Dhan Bahadur Shrestha, Jurgen Shtembari, Hirad Yarmohammadi, Talha Bin Farooq, Anuj Garg

**Affiliations:** 1 Division of Cardiology, Department of Internal Medicine, Bassett Medical Center, Cooperstown, New York, New York; 2 Division of Cardiology, Department of Internal Medicine, Carle Foundation Hospital, Urbana, Illinois; 3 Carle Illinois College of Medicine, Urbana, Illinois; 4 Division of Cardiology, Department of Medicine, New York-Presbyterian / Columbia University Irving Medical Center, New York, New York

**Keywords:** Ventricular fibrillation, Ventricular capture management, Short-long-short sequence, Personalized pacing, ICD pro-arrythmia

**Key Teaching Points**

• Automated capture management increases device longevity and smoothens the day-to-day clinical workflow in the device clinic.
• Ventricular capture management failure can lead to a short-long-short sequence, which can trigger polymorphic ventricular tachycardia.
• Personalized pacing programming is pivotal in selected cases with implantable cardioverter-defibrillator-mediated pro-arrhythmia.

## Introduction

With rapid advances in pacing and defibrillation systems, various fully-automated pacing algorithms are available from different vendors to improve workflow and patient safety.^1^, ^2^, ^3^, ^4^, ^5^, ^6^ Automated capture management (ACM) increases device longevity and reduces the need for frequent manual device interrogations and follow-ups.^6^^,^^7^ In the current-generation devices, automatic programming includes not only capture threshold testing but also assessment of sensing amplitude, battery voltage, lead impedance, mode switching, rate response, and atrio-ventricular interval adjustments, to name a few.^3^, ^4^, ^5^, ^6^, ^7^, ^8^

These features enable the miniaturization of pacemaker devices, longer device longevity, and safety in most instances.^3^, ^4^, ^5^ In this case, we report an extremely rare situation in which ventricular ACM failure led to a short-long-short (S-L-S) sequence and polymorphic ventricular tachycardia (PMVT), which subsequently degenerated to ventricular fibrillation (VF) and required multiple defibrillation shocks on separate occasions, successfully defibrillated by her cardiac resynchronization therapy defibrillator (CRT-D).

## Case summary

A 72-year-old woman with a history of recurrent ventricular arrhythmias was admitted after her CRT-D emitted an alert tone, raising concern for recent device therapies. Her medical history was notable for a prior VF storm requiring 63 appropriate implantable cardioverter-defibrillator (ICD) shocks, mixed ischemic and non-ischemic cardiomyopathy with recovered left ventricular (LV) ejection fraction 55%–60%, permanent atrial fibrillation status post atrioventricular node ablation, chronic obstructive pulmonary disease requiring home oxygen, and coronary artery disease with prior drug-eluting stent placement to the left anterior descending artery. She underwent implantation of a Medtronic CRT-D (Medtronic, Minneapolis, Minnesota) system in 2011, with multiple generator replacements, most recently in October 2022.

**Figure 1:**
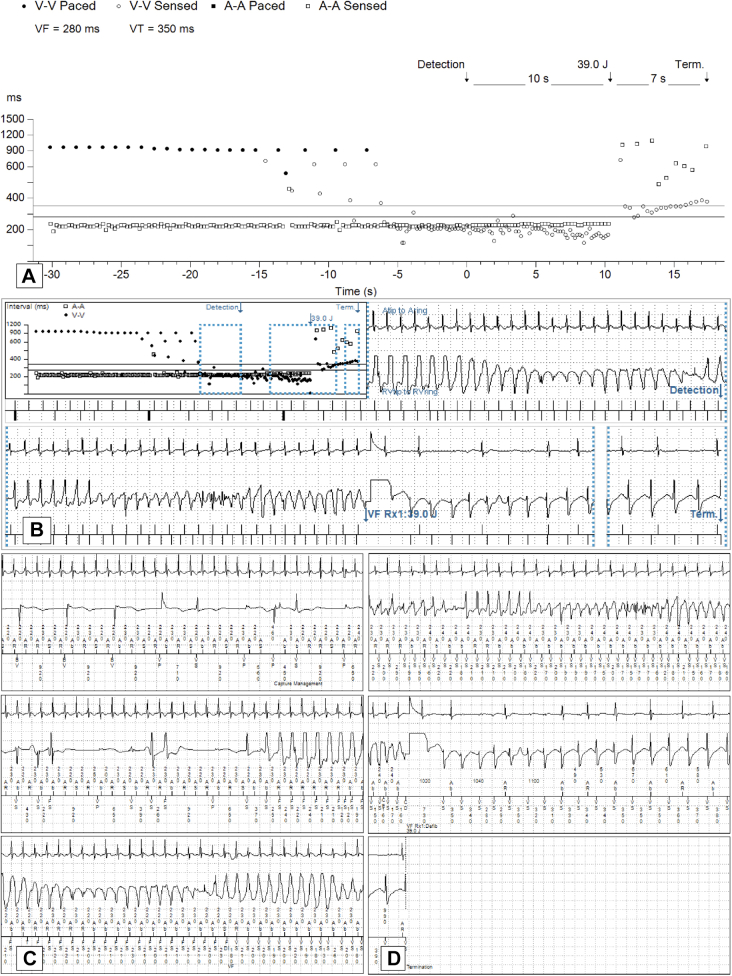
**A:** Interval plot showing a ventricular tachyarrhythmia. **B:** Arrhythmia summary. **C:** Atrial EGM showing underlying atrial flutter with ventricular EGM showing device performing capture management test with failure to capture resulting in significant bradycardia with slow escape ventricular beats and short-coupled PVCs eventually resulting in onset of polymorphic ventricular tachycardia in VF zone. **D:** Atrial EGM showing underlying atrial flutter with ventricular EGM showing persistent VF, which was appropriately sensed by the device and successful delivery of 39 J shock resulting in termination of atrial flutter and VF. EGM = electrogram; PVC = premature ventricular contraction; VF = ventricular fibrillation; VT = ventricular tachycardia.

The patient presented to the emergency department, reporting intermittent chest heaviness and concern that her device may have delivered shocks, although she did not clearly recall perceiving them. On interrogation, 3 recent ICD shocks were identified, occurring over the preceding 4 weeks; notably, these episodes were not simultaneous but occurred weeks apart (Figure 1 and Supplemental Figures 1 and 2). A retrospective review of stored electrograms revealed similar ACM-associated events in prior years, although there were instances of true ventricular arrhythmias not triggered by ACM, and the device appropriately detected and treated those episodes. At the time of presentation, she was hemodynamically stable and denied active chest pain, palpitations, syncope, or worsening dyspnea. Physical examination was unremarkable, with no evidence of volume overload or acute cardiopulmonary distress.

**Figure 2:**
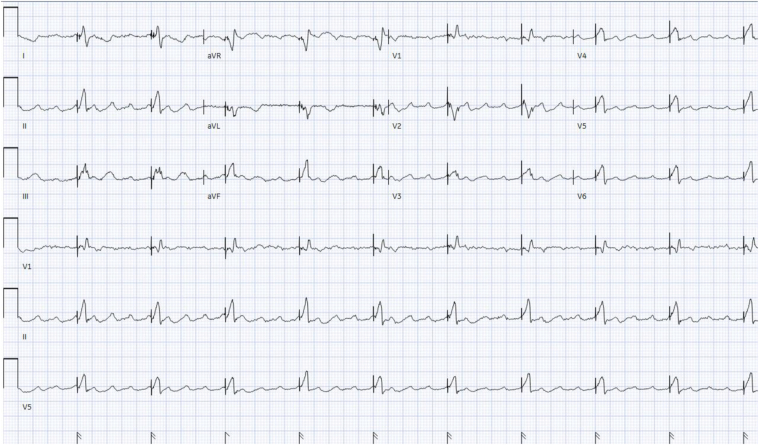
Electrocardiography showing a biventricular paced rhythm at 60 beats per minute with underlying atrial flutter.

Electrocardiography demonstrated a biventricular paced rhythm at 60 beats per minute (bpm) with underlying atrial flutter (Figure 2). Transthoracic echocardiography performed before admission revealed normal LV size and systolic function with an estimated ejection fraction of approximately 55%, mild concentric LV hypertrophy, and no significant valvular disease. Coronary angiography performed during the hospitalization showed non-obstructive coronary artery disease with hemodynamically insignificant lesions and normal LV filling pressures.

Device interrogation revealed a Medtronic Cobalt HF CRT-D (Medtronic) with stable lead impedances and preserved sensing and pacing thresholds (Table 1). The patient was ventricular pacing-dependent, with >99% biventricular pacing (Supplemental Figure 3). The device was programmed in standard CRT mode with DDDR pacing at a lower ventricular rate of 60 bpm using AdaptivCRT. The mode switch rate was set to 171 bpm, the upper tracking rate to 130 bpm, and the upper sensor rate to 120 bpm, with a paced atrioventricular (AV) delay of 130 ms and a sensed AV delay of 100 ms. Ventricular pacing was programmed from the left ventricle (LV) to the right ventricle (RV). For atrial tachycardia detection and therapies, the device was programmed to monitor >171 bpm with therapies turned off. For ventricular arrhythmias, 2 therapy zones were set: a VF zone for rates >214 bpm, with 1 burst anti-tachycardia pacing (ATP) followed by 40 J × 6 shocks; and a VT zone for ventricular rate between 171–214 bpm, with 3 bursts of ATP, 3 ramp of ATP sequences, followed by 40 J × 4 shocks. Additional detection enhancements were enabled for VT monitoring, atrial arrhythmias (atrial fibrillation, atrial flutter, sinus tachycardia, and supraventricular tachycardia), T-wave discrimination, and noise detection.

**Table 1:** Device or lead parameters measured at presentation

Parameters	Atrial (4076) lead	RV lead (6947)	LV lead (4196)
Pacing impedance	380 ohms	589 ohms	361 ohms
Defibrillation impedance		RV = 41 ohms, SVC = 55 ohms	
Pace polarity	Bipolar	Bipolar	LV tip to RV coil
Capture threshold	1.000 V @ 0.40 ms	0.625 V @ 0.40 ms	0.625 V @ 0.40 ms
Programmed amplitude/pulse width	1.50 V / 0.40 ms	2.00 V / 0.40 ms	2.00 V / 0.40 ms
Measured P/R wave	3.4 mV	10.6 mV	
Programmed sensitivity	0.30 mV	0.30 mV	

Atrial parameters are shown for completeness; however, atrial capture assessment was not clinically relevant given the patient’s history of atrial fibrillation and prior AV nodal ablation.

AV = atrioventricular; LV = left ventricle; RV = right ventricle; SVC = superior vena cava.

**Figure 3:**
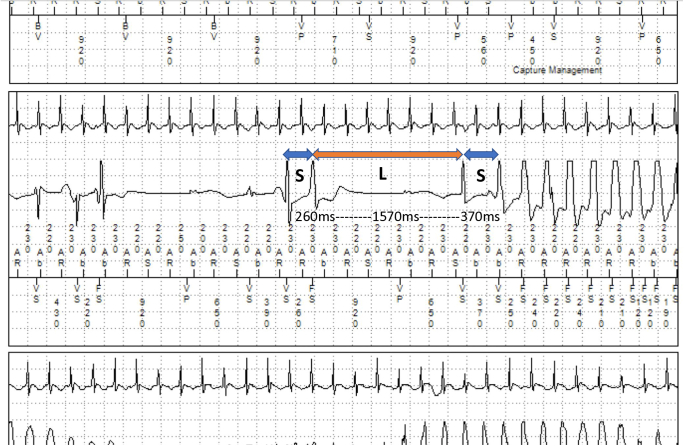
Zoomed section of failed ACM with S-L-S sequence initiating PMVT. Note the significant differences in short and long couple beats. ACM = automated capture management; PMVT = polymorphic ventricular tachycardia.

Review of stored electrogram (EGM) from all treated episodes demonstrated polymorphic ventricular tachycardia degenerating into VF, appropriately detected and terminated by high-energy shocks. Importantly, detailed analysis of the pre-event electrograms showed that each of the 3 arrhythmic episodes since the last interrogation was preceded by periods of reduced ventricular pacing output, associated with automatic ventricular capture management testing. During these intervals, transient loss of ventricular capture was observed, resulting in pacing pauses followed by premature ventricular complexes, forming an S-L-S sequence that triggered PMVT (Figures 1 and 3, and Supplemental Figures 1 and 2).

ACM was enabled for both right ventricular and LV leads. ACM operates without significant differences between single-ventricular lead systems and CRT systems; however, in CRT devices, LV capture assessment incorporates additional algorithmic checks, including atrioventricular conduction, interventricular conduction timing, stability assessments, and LV pacing threshold search. In this patient, the LV sensing response was programmed on but remains inactive during ACM testing. LV sensing was disabled during ACM to prevent cross-chamber sensing and ensure accurate threshold detection per programming feature. ACM in this device operates continuously rather than at fixed daily intervals.

No evidence of lead fracture, oversensing, or acute ischemia was identified. Given the consistent temporal association between capture management testing, transient loss of capture, and initiation of ventricular arrhythmias, the ventricular capture management feature was disabled. The device was reprogrammed to fixed pacing outputs with an increased safety margin. Following these changes, no further ventricular arrhythmias or ICD therapies were observed during the remainder of the hospitalization. Intravenous amiodarone was initiated empirically in the emergency department and subsequently transitioned to oral therapy.

## Discussion

ACM in pacemakers was conceptualized soon after the first pacemaker was implanted in humans; however, its practical use was initially limited by challenges in reliable capture detection.^4^ The Medtronic capture management algorithm has been designed to function effectively for right atrial, right ventricular, and LV capture in CRT devices.^9^

Right ventricular ACM algorithms operate by sensing the electrical response evoked by pacing and analyzing the slew rate to confirm myocardial capture. In contrast, in CRT systems, LV capture management incorporates additional cross-chamber and conduction-based assessments, including LV-RV conduction timing and stability algorithms.^9^ LV sensing is typically disabled during testing to avoid cross-chamber signal contamination. Although early-generation LV-ACM algorithms were considered less reliable than RV-based systems, a study using algorithmic checks for AV conduction, LV-RV conduction, stability assessments, and the LV pacing threshold in a Medtronic CRT-D found it to be highly reliable, safe, and accurate.^10^ Across currently available devices, automated functions, including ACM, generally perform well, with appropriate automatic modulations of pacing output. These advancements have improved workflow efficiency and overall safety in contemporary device management.^1^^,^^2^^,^^4^, ^5^, ^6^, ^7^, ^8^, ^9^

Despite increasingly complex algorithms, these systems perform well in most instances; however, important caveats remain. These include difficulty in fully understanding all automatic modulations because of their complexity, and the potential for unintended events resulting from faulty device sensing during pacing threshold testing.^9^^,^^11^

In pacemaker-dependent patients, ventricular ACM malfunction can lead to catastrophic loss of ventricular capture, as demonstrated in our case. Conversely, malfunction of ventricular ACM can also result in unnecessarily high pacing output.^11^ By design, right atrium and RV ACM algorithms are unable to increase pacing output beyond programmed upper safety limits (>5.0 V and 1 ms pulse width).^9^ In our patient, who was pacing-dependent, ventricular pacing during ACM threshold testing resulted in a transient loss of capture because of the device’s inability to detect capture failure. Each test pacing sequence is typically preceded by 3 support pacing cycles and includes backup pacing with programmed amplitude at higher pulse widths (eg, 1 ms) to prevent pauses; however, in this case, intermittent failure during threshold testing still resulted in clinically significant pauses. These pauses were followed by a slow ventricular escape rhythm and short-coupled premature ventricular complexes, creating a S-L-S sequence that triggered subsequent PMVT. Fortunately, in all 3 episodes, the device appropriately detected VF and successfully terminated the arrhythmia with high-energy shocks.

Importantly, this phenomenon was not a new finding in our patient, as a retrospective review demonstrated similar ACM-associated events in prior years, and there was no evidence of lead malfunction, with stable sensing, impedance, and capture thresholds throughout the follow-up period.

In patients with an ICD, as in our case, ICD-related pro-arrhythmia is most commonly attributed to pacing-facilitated S-L-S sequences that trigger PMVT.^12^^,^^13^ Lower-rate programming (DDD/VVI) and algorithms such as Managed Ventricular Pacing (designed to minimize ventricular pacing) may permit an S-L-S sequence, and subsequent PMVT. In contrast, rate-smoothing algorithms may help prevent such events. Susceptible individuals may therefore require individualized pacing programming; however, identifying these patients prospectively in clinical practice remains challenging.^12^^,^^13^ To our knowledge, S-L-S sequences and PMVT mediated specifically by capture-management algorithms have not been previously reported in the literature, although such pro-arrhythmic potential was reported to be plausible mechanistically.^13^

## Conclusion

In individuals with an ICD, ACM failure may lead to life-threatening ventricular arrhythmia mediated by a S-L-S sequence. A thorough understanding of automated device functions in current-generation systems, along with individualized pacing programming, is essential in selected cases of ICD-mediated pro-arrhythmia.

## Disclosures

The authors have no conflicts of interest to disclose.

## Footnotes

Appendix Supplementary data associated with this article can be found in the online version at https://doi.org/10.1016/j.hrcr.2026.03.026.
