# Supplementary material for: Automated capture management failure leading to ventricular fibrillation: Is automatic capture management for all or personalized pacing programming?
Source: HeartRhythm Case Rep. 2026 Apr 7;12(7):744–8. doi: 10.1016/j.hrcr.2026.03.026 (PMC13379351; doi:10.1016/j.hrcr.2026.03.026)
Supplement: Supplementary File [file mmc1.docx]

Supplementary file

**
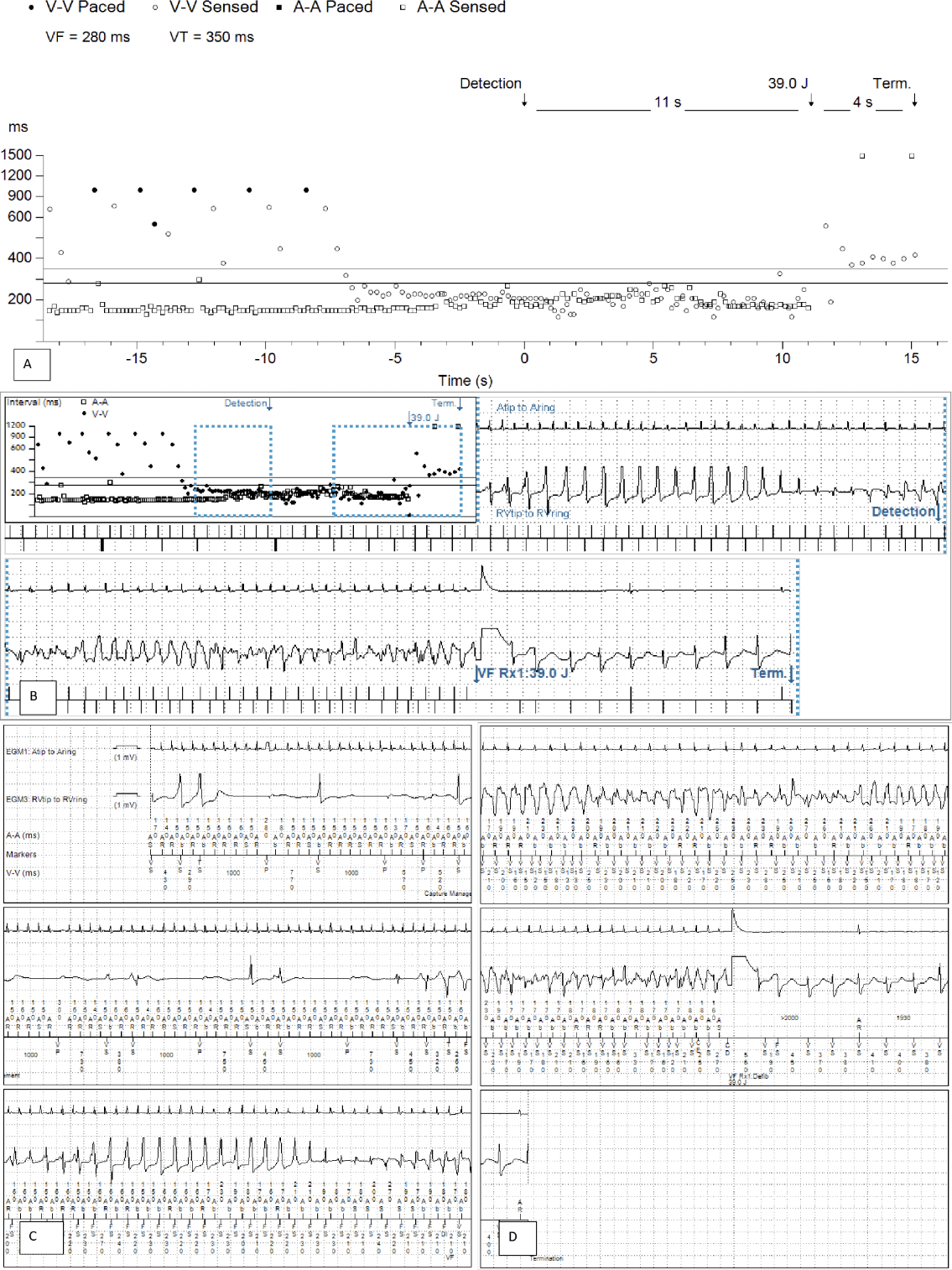
**

**Figure 1.** Panel A. Interval plot showing a ventricular tachyarrhythmia (A-A: AA intervals; V-V: VV intervals: VF =ventricular fibrillation; VT = ventricular tachycardia). Panel B. Arrhythmia summary, Panel C. Atrial EGM showing underlying atrial flutter with ventricular EGM showing device performing capture management test with failure to capture resulting in significant bradycardia with slow escape ventricular beats and short coupled PVCs eventually resulting in onset of polymorphic ventricular tachycardia in ventricular fibrillation zone Panel D. Atrial EGM showing underlying atrial fibrillation with ventricular EGM showing persistent ventricular fibrillation, which was appropriately sensed by the device and successful delivery of 39J shock resulting in termination of tachycardia

**
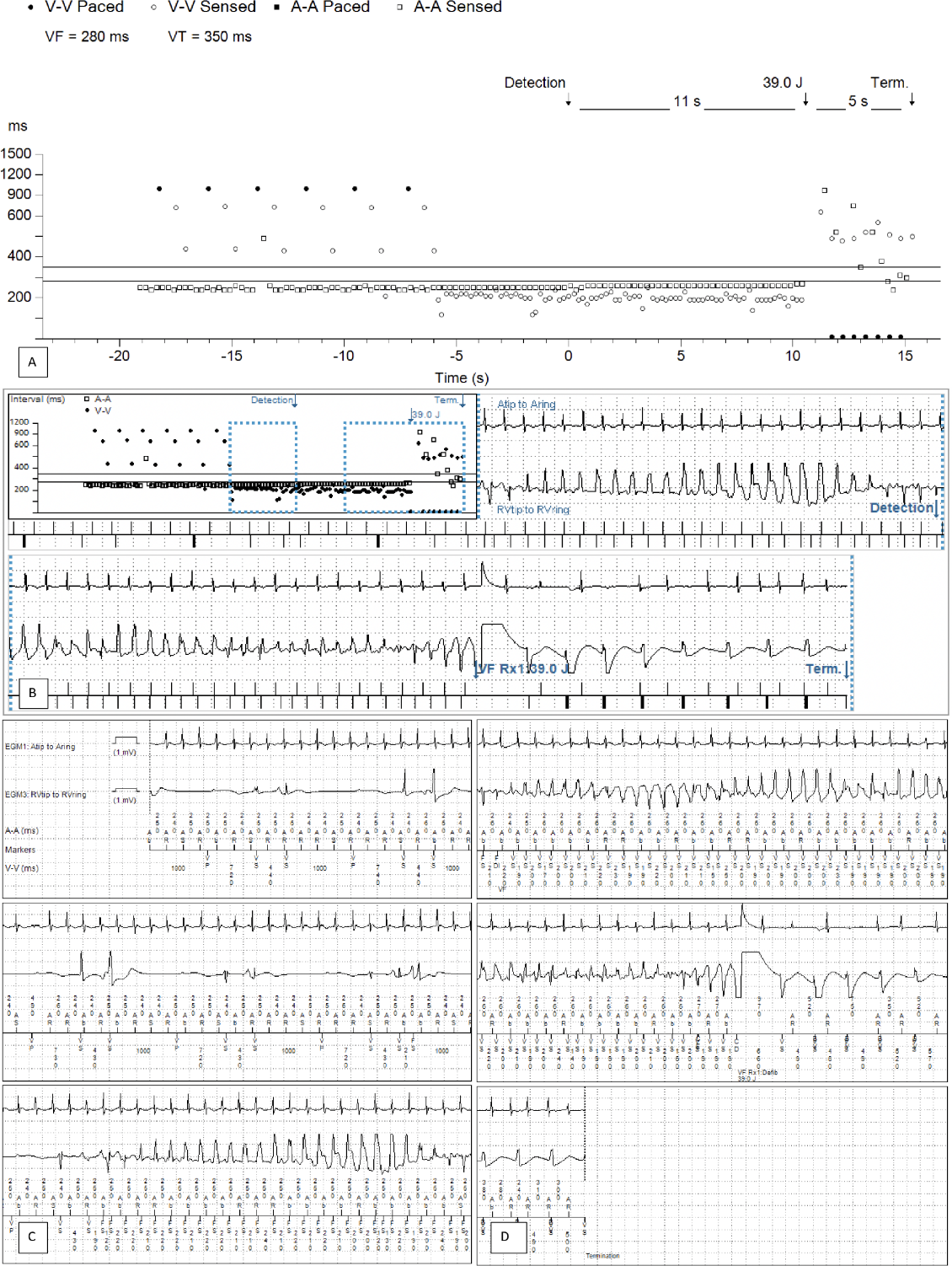
**

**Figure 2.** Panel A. Interval plot showing a ventricular tachyarrhythmia (A-A: AA intervals; V-V: VV intervals: VF =ventricular fibrillation; VT = ventricular tachycardia). Panel B. Arrhythmia summary, Panel C. Atrial EGM showing underlying atrial flutter with ventricular EGM showing device performing capture management test with failure to capture resulting in significant bradycardia with slow escape ventricular beats and short coupled PVCs eventually resulting in onset of polymorphic ventricular tachycardia in ventricular fibrillation zone. Panel D. Atrial EGM showing underlying atrial fibrillation with ventricular EGM showing persistent ventricular fibrillation, which was appropriately sensed by the device and successful delivery of 39J shock resulting in termination of tachycardia

**
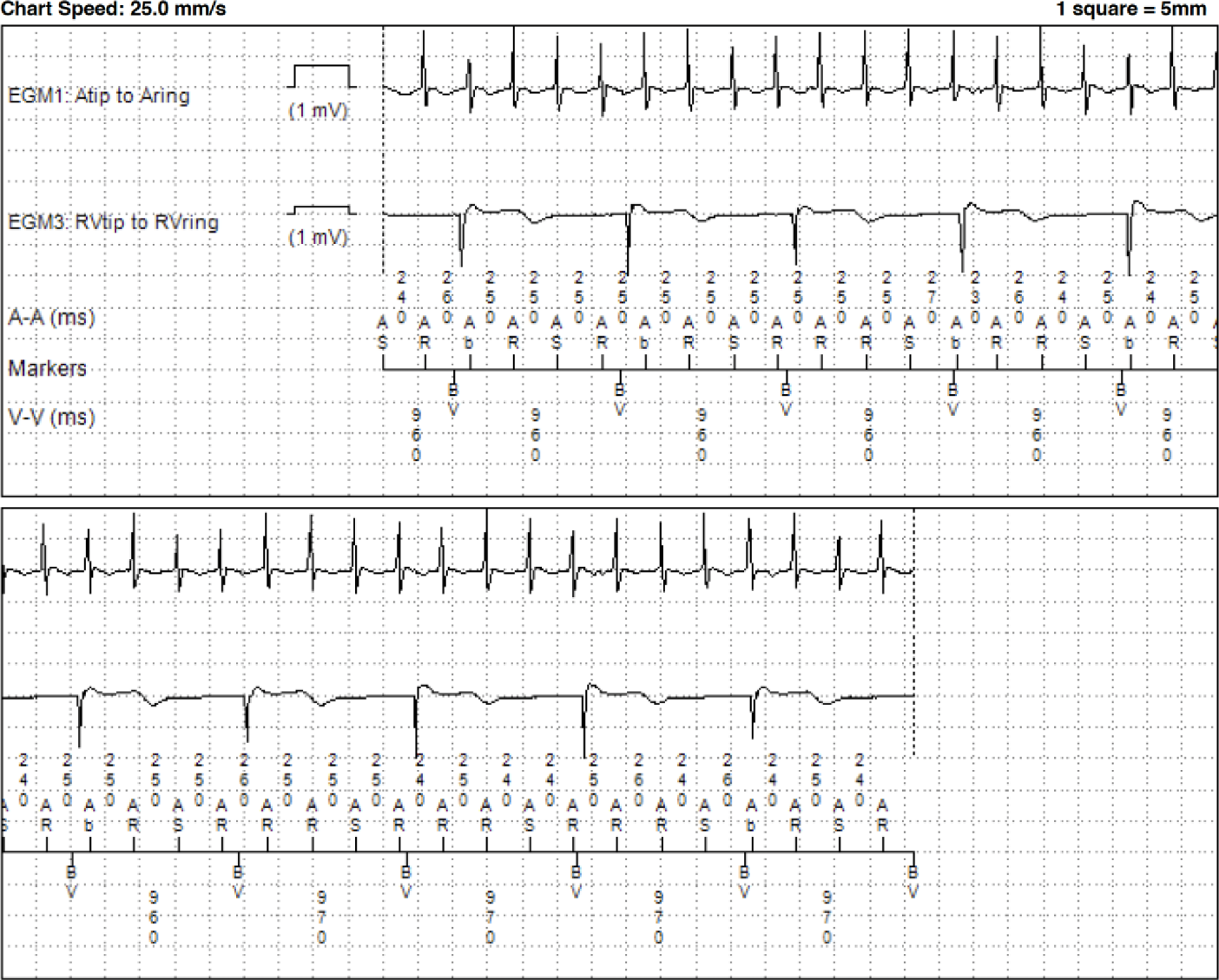
Figure 3**. Baseline EGM: Atrial EGM showing underlying atrial flutter, with ventricular EGM showing ventricular pacing
